# RELATIVE AND ABSOLUTE METABOLIC COST OF DAILY PHYSICAL ACTIVITIES ACROSS THE LIFESPAN

**DOI:** 10.1093/geroni/igad104.3803

**Published:** 2023-12-21

**Authors:** Todd Manini, Eileen Handberg, Mamoun Mardini, Bhanu Sandesara, Ramon Casanova, Duane Corbett, Amal Wanigatunga

**Affiliations:** University of Florida, Gainesville, Florida, United States; University of Florida, Gainesville, Florida, United States; University of Florida, Gainesville, Florida, United States; University of Florida, Gainesville, Florida, United States; Wake Forest University, Winston Salem, North Carolina, United States; Axogen, Gainesville, Florida, United States; Johns Hopkins School of Public Health, Baltimore, Maryland, United States

## Abstract

Americans spend over 90% of their activity related energy expenditure performing common daily activities. However, there is a gap in knowledge pertaining to potential age-related differences in the metabolic cost of performing these activities which diminishes proper physical activity prescription. We hypothesized age differences in both the absolute and relative metabolic cost of performing common daily physical. Participants (n=248, age: 20-94 yrs) performed 33 activities (e.g. dressing, walking, making a bed, gardening, computer work) in a standardized setting. A portable indirect calorimeter was worn for approximately 6-10 minutes to achieve steady-state metabolic rate. A graded exercise treadmill test was performed to quantify peak metabolic rate. A metabolic equivalent (MET as a function of 3.5 milliliter•min-1•kg-1) and percentage of peak were used to express absolute and relative metabolic rate, respectively. Watching television (1.0+/-0.36 METs; 15.6+/-5.7% of peak) produced the lowest and climbing stairs (6.2+/-2.2 METs; 84.8+/-17.0% of peak) elicited the highest metabolic rate. Results from mixed effect regression models adjusting for health conditions, demographics and anthropometry showed no association between absolute MET and age. However, when expressed relative to peak, adjusted models showed a strong association with age (0.66+/-0.33% per year, p=0.006). As an example, adults < 40 yrs old worked at 34.1+/-14.8% of peak during a leisurely walk while those 80+ years old worked at 64.2+/-16.3%. Aging is associated with a substantial increase in the relative metabolic cost of performing common daily physical activities. These results could have implications on prescribing physical activity older adults to achieve higher fitness.

